# MicroRNA functions in osteogenic differentiation of periodontal ligament stem cells: a scoping review

**DOI:** 10.3389/froh.2025.1423226

**Published:** 2025-01-31

**Authors:** Pirawish Limlawan, Anjalee Vacharaksa

**Affiliations:** ^1^ Department of Oral Medicine, Faculty of Dentistry, Chulalongkorn University, Bangkok, Thailand; ^2^ Center of Excellence and Innovation for Oral Health and Healthy Longevity, Faculty of Dentistry, Chulalongkorn University, Bangkok, Thailand; ^3^ Research Unit on Oral Microbiology and Immunology, Faculty of Dentistry, Chulalongkorn University, Bangkok, Thailand; ^4^ Department of Microbiology, Faculty of Dentistry, Chulalongkorn University, Bangkok, Thailand; ^5^ Master of Science Program in Geriatric Dentistry and Special Patients Care, Faculty of Dentistry, Chulalongkorn University, Bangkok, Thailand

**Keywords:** microRNA, periodontal ligament stem cells, osteogenic differentiation, bone engineering, tissue regeneration

## Abstract

This scoping review aimed to describe the differential microRNA (miRNA) functions in osteogenic differentiation of periodontal ligament stem cells (PDLSCs), and then analyze the potential of applying PDLSCs and miRNAs in bone regeneration. The databases of PubMed, Google Scholar and EBSCO search were performed by the 4 themes, including periodontal ligament stem cells, miRNA, osteogenic differentiation, and tissue regeneration. The original articles described miRNA functions in osteogenic differentiation of PDLSCs were identified and selected for content analyze. The articles suggested that PDLSCs have high potential in bone regeneration because of their multipotency and immunomodulation. PDLSCs are conveniently accessible and obtained from extracted teeth. However, recent evidence reported that PDLSCs of various origins demonstrate differential characteristics of osteogenic differentiation. Exosomal miRNAs of PDLSCs demonstrate a regulatory role in tissue regeneration. The properties of PDLSCs associated to miRNA functions are altered in differential microenvironmental conditions such as infection, inflammation, high-glucose environment, or mechanical force. Therefore, these factors must be considered when inflamed PDLSCs are used for tissue regeneration. The results suggested inflammation-free PDLSCs harvested from the middle third of root surface provide the best osteogenic potential. Alternatively, the addition of miRNA as a bioactive molecule also increases the success of PDLSCs therapy to enhance their osteogenic differentiation. In conclusion, Exosome-derived miRNAs play a key role in PDLSCs osteogenic differentiation during tissue regeneration. While the success of PDLSCs in tissue regeneration could be uncertain by many factors, the use of miRNAs as an adjunct is beneficial for new bone regeneration.

## Introduction

Periodontitis is an inflammatory disease of the periodontal tissue that causes bone destruction. Periodontal defects could be regenerated using cell therapy (1). The stem cell potential for therapy depends on cell origins such as bone marrow, adipose tissue, embryo, umbilical cord, or dental origin (2). Periodontal ligament stem cells (PDLSCs) are pluripotent and able to repair the advanced periodontal defect (3). PDLSCs therapy was demonstrated to regenerate periodontal tissue in animal models and one human trial. In beagle dogs, PDLSCs transplantation in the advanced periodontal lesion was demonstrated without bone scaffold (3). Moreover, transplantation of healthy PDLSCs regained the height of alveolar bone more than HA/TCP scaffold alone in a periodontitis swine model (4, 5). In the human randomized-control trial, PDLSCs combined with bovine-derived bone graft materials improved the alveolar bone height in periodontal intrabony defects (6). When transplanted, PDLSCs induce favorable host immune responses in the surrounding tissue (7) by inhibiting T cell proliferation (8). These findings suggest a benefit of PDLSCs therapy, while a scaffold or bioactive molecules are essential addition in a critical-sized bone defect to improve outcomes (9). In some cases, hopeless teeth were used to provide multipotent PDLSCs for tissue regeneration. Osteogenic potential is therefore the key to success (10), but PDLSCs from different origins might be varied in osteogenic potential. The recent evidence also suggested that inflammation or aging could affect osteogenic potential.

Recent studies suggested that the success of PDLSCs therapy is promising but this approach may have limitation on cell availability and how to maintain osteogenic potential of implanted cells. The question remains whether adding bone scaffold or bioactive molecules increases the success of PDLSCs transplantation. MicroRNAs (miRNA) are single stranded, 20–30 nucleotides long, non-coding RNAs, that post-transcriptionally regulate gene expression by partially or fully complementary pairing with their targeted mRNAs for translation blockage or degradation (11). MiRNAs can be secreted through an exosome pathway into extracellular compartment before transported to target cells (12). Extracellular miRNAs could mediate cell communication while miRNAs function to control gene expression and protein translation intracellularly (13). MiRNAs have been reported to play a key role within exosomes derived from mesenchymal stem cells that potentially regulate many cellular signaling pathways. These findings therefore suggested miRNAs as the potent mechanism of the PDLSCs to achieve bone regeneration. The objectives of this scoping review are to describe the differential osteogenic potential of PDLSCs in healthy or inflamed conditions, and PDLSCs-associated miRNAs relating to their functions in osteogenic differentiation. Since the success of PDLSCs therapy may be hindered by local environment, the potential application of miRNAs to improve PDLSCs properties for desired outcomes in clinical settings is discussed.

## Methods

### Protocol design

Methods for this study were developed based on the scoping review methodology presented by Arksey and O'Malley, 2005 (14) and Levac et al., 2010 (15). According to this framework, five stages were undertaken: (1) identifying the research question; (2) identifying relevant studies; (3) selecting studies; (4) charting the data; and (5) collating, summarizing and reporting the results. The protocol of this study was registered in OSF Preregistration (https://doi.org/10.17605/OSF.IO/KE4XU) before data analysis.

### Stage 1: Identifying the research question

The main research question focuses on understanding the differential osteogenic potential of PDLSCs in healthy or inflamed conditions and PDLSCs-associated miRNAs relating to their functions in osteogenic differentiation. For these purposes, the following questions guided this review.

1. Which characteristics of PDLSCs determine clinical success in application? Did PDLSCs from different origins share similar results in osteogenic potential? How to obtain desired PDLSCs function?
2. Which miRNAs associate with the desired PDLSCs osteogenic potential?
3. How differential miRNA profile affects PDLSCs osteogenic potential under inflammation conditions?
4. What are potential applications of miRNAs to improve PDLSCs properties in clinical settings?

### Stage 2: Identifying relevant studies

**Figure 1:**
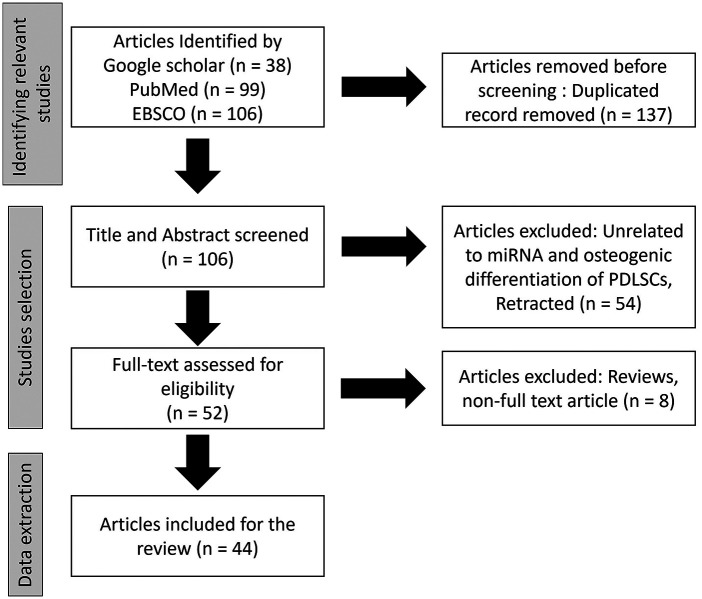
Flow chart illustration the article selection.

To identify relevant studies to this review, databases included electronic databases of the published literature including Google Scholar, PubMed, and EBSCO (Figure 1). The search was conducted on published literature from year 2000 to the present. The language was limited to English. To ensure that all relevant information was captured, hand-search on all reference lists of included studies was performed to identify additional studies of relevance. Search terms were determined with input from the research team and collaborators. Database and other searches combined terms from four themes: periodontal ligament stem cells (PDLSCs), microRNAs (miRNA), osteogenic differentiation, and tissue regeneration. Terms were searched in the title and/or abstract and subject headings as appropriate. The search strategy was developed by an experienced research librarian and co-authors. The results were exported to Endnote and later exported to Covidence software (16) for title and abstract screening. The original articles described miRNA functions in osteogenic differentiation of PDLSCs were identified and selected for content analyze. Reviews and non-full text articles were excluded. were To investigate the experience or meaningfulness of a particular phenomenon, a description of the PICo (population, phenomenon of interest, and context) (17) elements is outlined below to guide the screening and identification of relevant studies.

For the inclusion and exclusion criteria, the searches population was focused on the human PDLSCs extracted from human periodontal ligament tissue. Therefore, other types of stem cells, such as bone marrow-derived or dental pulp-derived cells, were excluded. For the phenomenon of interest and its context, the search identified PDLSCs osteogenic potentials reported in healthy, or various inflammatory conditions. Therefore, osteogenic differentiation induced by other bioactive molecules, such as BMP were excluded (Supplementary Table 1). The potential of PDLSCs was related to their miRNA functions to induce osteogenic differentiation.

### Stage 3: Selecting studies

Title and abstract screening were done by two independent reviewers to select studies related to the PICo format as described above. Understanding of the inclusion and exclusion criteria was calibrated through a pilot screening of a few studies adhering strictly to the PICo criteria. Next, the full-text screening was done by two independent reviewers selecting studies according to the inclusion criteria. Studies with no full-text, or meet the exclusion criteria, were excluded. Data were extracted by two independent reviewers and inter-rater reliability will be discussed against the themes. The results of the search were reported and presented in a PRISMA flow diagram. Any disagreements between the reviewers were discussed and resolved through consensus.

### Stage 4: Extracting data

Key information was collected from the relevant studies including publication year, authors and affiliation, title, research questions, objectives, population, methods, miRNAs, context (healthy or inflammation), and study findings. The data were recorded in a table and outcomes were categorized. To ensure the validity of the process, the table was piloted and tested against a few studies by coauthors. Two independent reviewers then extracted the data, and disagreements were discussed among the team members. A quality appraisal of the primary studies included in the review was assessed.

### Stage 5: Collating, summarizing and reporting the outcomes

According to the proposed questions, the scoping review provided an aggregated synthesis of the evidence on the four components including (1) Characteristics of Periodontal Ligament Stem cells from different sources; (2) MicroRNA functions during osteogenic differentiation of PDLSCs; (3) MicroRNA functions in PDLSCs under inflammation conditions; (4) Current application of stem cells and miRNAs in orofacial bone tissue engineering. The data collected from the included studies was summarized narratively. The scoping review also present our experiences and recommendations on the potential use of stem cells and miRNAs as bioactive molecules in bone regeneration.

## Results

### Selected study for reviews

From the databases of Google Scholar, PubMed, and EBSCO, total 243 articles were identified. After duplicated record removal, 137 articles were removed. Total 106 article were screen by title and abstract and 54 unrelated articles were exclude. Full-text assessment for eligibility was done in 52 articles with the exclusion of 8 articles due to being reviews and non-full text article. Finally, 44 of the articles were included in this review.

### Characteristics of periodontal ligament stem cells from different sources

Periodontal ligament (PDL) tissue is composed of heterogeneous cell populations including fibroblasts, endothelial cells, osteoblasts, cementoblasts, and multipotent stem cells. The periodontal tissue is always exposed to oral fluid filled with microorganisms which contribute to the host immune response and PDLSCs osteogenic differentiation (18). The PDLSCs, in the remnants of PDL retained in extraction sockets (19), or transplanted PDLSCs in bone defect (20), may contribute as osteoprogenitor cells for bone regeneration. The putative stem cells in PDL tissue, or PDLSCs, derived from the epithelial cell rests of Malassez, which have a role in the maintenance and regeneration of periodontal tissues (21, 22). In general, PDLSCs demonstrated a higher proliferating rate, stronger collagen fiber formation, but lower osteogenic differentiation in comparison to bone marrow-derived mesenchymal stem cells (23). Nonetheless, PDLSCs retain a high potential in cell therapy concerning their availability upon tooth extraction. One of the PDLSCs advantages is the cells harvested from extracted tooth to be used within the same person. PDL from orthodontic-extracted, or uninfected teeth, provides healthy cells (24, 25), those can be expanded in culture while retaining their multipotent property for transplantation (3, 26). However, it is unclear whether inflamed tissue from periodontal hopeless teeth provides adequate potential. Therefore, it is important to consider the factors affecting PDLSCs osteogenic differentiation. The characteristics and origins of PDLSCs that could determine clinical success in its osteogenic potential and clinical application should be clarified.

In healthy condition, stem cells extracted from PDL of the middle or apical third of the root demonstrated different cell surface markers and characteristics. PDL of the apical third includes stem cells from periapical follicles (SCAP) (27). SCAP are characterized by a specific marker, CD24 (28), and they are likely to be a source of primary odontoblasts of root dentin (29). In contrast to SCAP, PDL from the middle third was preferable to be continued in cell culture (30) as they exhibit surface markers, including STRO-1, CD146, CD90, CD105 and CD44 (27, 31). Expression of CD24 correlates with multi-lineage differentiation ability but inversely correlates with self-renewal ability of SCAP (32). These markers represent the cells with stemness in these mesenchymal stem cells (33).

Since the source of PDLSCs could be from hopeless teeth, it was questioned whether aging or inflammation affected its potential. The proliferation and the differentiation capacity of PDLSCs inversely correlated to age. In the study by Zhang et al., PDLSCs obtained from the 56- to 75-year-old donors significantly decreased pluripotency compared to the 16- to 55-year-old donors (34). Li et al. demonstrated that immunosuppressive ability of aged PDLSCs decreased (35). Interestingly, the conditioned medium from PDLSCs of younger donors enhanced differentiation and proliferation ability of aged PDLSCs suggesting that the multipotency of an old PDLSCs may be restored by the modulators released from the PDLSCs of younger donors (36). It is therefore possible to culture PDLSCs from the aged teeth, however the stemness of cells may be reduced (31, 34, 35). Whether the inflamed tissue could be a source of stem cell transplantation remains controversial. PDLSCs from periodontitis hopeless teeth have proliferation and migration potential (31), but they demonstrated lower osteogenic differentiation and immunomodulation (37). The study by Xu et al., reported that osteogenic differentiation of PDLSCs may be impaired in inflammatory condition due to P2X7 receptor downregulation (38). Collectively, PDLSCs of inflamed tissue could be expandable, but the osteogenic potential of PDLSCs appears compromised due to inflammation.

### MicroRNA functions during osteogenic differentiation of PDLSCs

In the search for miRNAs associate with the desired PDLSCs osteogenic potential, the data indicated several major cell signaling pathways, including canonical Wnt/β-catenin (39–41), Smad (42, 43), MAPK (44) and epigenetic level-Histone deacetylase (HDAC)6 (45), involved osteogenic differentiation of PDLSCs.

#### Wnt/β-catenin pathway

The Wnt proteins are the key molecules to regulate stem cell functions and have a role in bone formation (40, 46). The canonical or non-canonical pathways are categorized by the role of β-catenin. The canonical Wnt signaling requires a function of β-catenin in contrast to the non-canonical pathway (47). Several miRNAs were reported to contribute in the canonical Wnt/β-catenin pathway of PDLSCs osteogenic differentiation (Table 1). Yet there was no report of miRNA function involved in the non-canonical pathway. To initiate signaling, Wnt proteins bind Frizzled (Fz) receptor family and co-receptors such as the low-density-lipoprotein-related protein 5/6 (LRP5/6). In the canonical pathway, β-catenin is translocated into nucleus and interact with T-cell factor/lymphoid enhancer factor (Lef/Tcf) transcription factor for osteogenic differentiation. The decrease of Wnt signal was reported with miRNA-758, miRNA-214, and miRNA-17 expression or by forming a destruction complex. When miRNA-758 is expressed, Notch receptor 2 (Notch2) is targeted and downregulated. As a result, both Wnt and β-catenin protein decreased, and osteogenic differentiation was inhibited (41). MiRNA-214 targets CTNNB1 mRNA, encoding β-catenin protein (39) and the Activating Transcription Factor 4 (ATF4), which is the transcription factor of β-catenin in PDLSCs (71), leading to suppression of osteogenic differentiation (49). While MicroRNA-17 targets the transcription factor, Tcf3, of the β-catenin signaling (48), resulted in the inhibition of osteogenic gene expression. In addition, cytoplasmic β-catenin level is controlled by forming a β-catenin destruction complex. Casein Kinase and glycogen synthase kinase 3 (GSK3) mediate phosphorylation of β-catenin within the destruction complex for ubiquitination (46). However, miRNA-374a targets adenomatous polyposis coli (APC), an essential component of the APC/Axin/GSK3/β-catenin destruction complex to increase cytosolic β-catenin. Expression of miRNA-374a resulted in an increase of β-catenin in the nuclear compartment to support osteogenic gene expression (50). Taken together, the expression of these miRNAs plays a role in the canonical Wnt/β-catenin pathway to regulate osteogenic differentiation of PDLSCs.

**Table 1:** MiRNAs involvement in osteogenic differentiation of PDLSC.

Pathway	MicroRNA	Effect	Target	References
Wnt/β-catenin pathway	miRNA-17	Suppress	TCF3: transcription factor of Wnt/β-catenin pathway	(48)
	miRNA-214	Suppress	Activating transcription factor 4	(49)
	miRNA-214	Suppress	β-catenin gene CTNNB1	(39)
	miRNA-374a	Promote	APC in β-catenin destruction complex	(50)
	miRNA-758	Promote	Notch2 in Wnt/β-catenin pathway	(41)
Smad pathway	miRNA-7	Suppress	GDF5 promoter of R-Smad1/5/8 phosphorylation	(44)
	miRNA-21	Suppress	Smad5	(42)
	miRNA-24-3p	Suppress	Smad5	(51)
	miRNA-106a-5p	Suppress	BMP2	(52)
	miRNA-222-3p	Suppress	Smad2 and 7	(43)
	miRNA-4781-3p	Suppress	Smad5	(53)
NF-κB	miRNA-125b	Suppress	NKIRAS2 in NF-κB Pathway	(54)
Histone deacetylase	miRNA-22	Promote	Histone deacetylase 6	(45)
	miRNA-22-3p	Promote	SIRT1	(55)
	miRNA-153-3p	Suppress	Lysine demethylase 6A	(56)
	miRNA-383-5p	Promote	Histone deacetylase 9	(57)
Other	Let-7b	Suppress	Collagen triple helix repeat containing 1	(58)
	miRNA-10a-5p	Suppress	Brain-derived neurotrophic factor	(59)
	miRNA-21, 101	promote	Periodontal ligament associated protein 1	(60)
	miRNA-30c	Suppress	Osteocalcin	(61)
	miRNA-152-3p	Suppress	Integrin alpha 5	(62)
	miRNA-155-5p	Suppress	E26 transformation specific-1	(63)
	miRNA-181b-5p	Promote	PTEN in AKT signaling pathway	(64)
	miRNA-184	Suppress	Nuclear factor I-C	(65)
	miRNA-218	Suppress	RUNX2	(66)
	miRNA-543	Promote	Transducer of ERBB2,2	(67)
	miRNA-589-3p	Promote	Activating transcription factor 1	(68)
	miRNA-874-3p	Suppress	Vascular endothelial growth factor A	(69)
	miRNA-2861	Promote	Unspecified	(70)

#### Smad pathway

Complete activation of the Smad pathway, the key signaling pathway in growth and development, results in transcription of osteogenic genes (72). Smad proteins are receptors of the transforming growth factor beta (TGF-B) superfamily. When the ligand binds transmembrane receptor serine/threonine kinases, R-Smad is phosphorylated and released to form a heterotrimeric complex which is then translocated into the nucleus. Many miRNAs have regulatory functions in the Smad signaling pathway including miRNA-7, miRNA-21, and miRNA-222-3p (Table 1). The expression of MiRNA-7 suppresses osteogenic differentiation of PDLSC by targeting growth different factor5 (GDF5)-specific mRNA, which functions to promote phosphorylation of R-Smad including Smad1, 5 and 8 (44). MiRNA-21, -4781-3p, -24-3p suppresses osteogenic differentiation by targeting the Smad5 gene (42, 51, 53). MiRNA-222-3p suppresses osteogenic differentiation by targeting Smad2 and Smad7 which are known to promote osteogenic differentiation (43). MiRNA-106a-5p suppresses osteogenic differentiation by targeting bone morphogenetic protein 2 (BMP2), an important ligand for Smad pathway regulating the differentiation (52).

#### Other pathways

During osteogenic differentiation, miRNAs involve in the nuclear factor kappa light chain enhancer of activated B cells (NF-κB) pathway, The p38 mitogen-activated protein kinase (MAPK) pathway, epigenetic regulation, and other undefined mechanisms. To promotes osteogenic differentiation of PDLSCs through NF-κB signaling, miR-125b targets the NF-κB inhibitor interacting RAS-like 2 (NKIRAS2) (54), while miRNA-146a (73), possibly through the downregulation of NF-κB (74).

In osteoblasts, the MAPK signal cascades positively regulate the transcriptional activity of distal-less homeobox 5 (DLX5), Runt-related transcription factor 2 (RUNX2) and Osterix (OSX) that are involved in PDLSC differentiation (75). While the expression of miRNA-7 results in reduction of osteogenic differentiation by targeting the growth differentiation factor 5 (GDF5) required for p38 phosphorylation in the signal activation (44). MiRNA-218 targets RUNX2, the master transcription factor for osteogenic differentiation, which leads to suppression of osteogenic differentiation in PDLSCs (66). MiRNAs also regulate Histone deacetylases in osteogenic differentiation. MiRNA-22 and miRNA-383-5p targets histone deacetylase 6 (HDAC6) and 9 (HDAC9) respectively, and therefore promote osteogenic differentiation (45, 57). MiRNA-22-3p inhibits Sirtuin 1 Silent mating Type information regulation 2 homolog 1 (SIRT1), a class III histone/protein deacetylase, that mediated bone resorption (55), resulting in an increase of osteogenic differentiation (76). However, miRNA-153-3p suppresses osteogenic differentiation by targeting Lysine demethylase 6A (KDM6A) the Histone three lysine 27 (H3K27) demethylase (56).

Downregulation of some miRNAs, including miRNA-Let-7b, -155-5p, -10a-5p, -152-3p, -874-3p, -184 and miRNA-30c occurs during osteogenic differentiation. MiRNA-Let-7b targets Collagen triple helix repeat containing 1 (CTHRC1) the promoter of osteogenic differentiation (58). CTHRC1 is known to be a protein secreted by mature osteoclasts, to stimulate osteogenesis as a coupling factor for bone resorption to formation (77). MiRNA-155-5p targets E26 transformation specific-1 (ETS1) (63) which is a transcription factor known to be involved in osteoblast differentiation (78). MiRNA-10a-5p targets a growth factor brain-derived neurotrophic factor (BDNF) which is also released by PDLSCs for osteogenic differentiation (59). MiRNA-152-3p targets integrin alpha 5 (ITGA5) (62) which alters PDLSCs cytoskeleton and cell cycle during osteogenic differentiation (79). MiRNA-874-3p targets vascular endothelial growth factor A (VEGFA) (69) an enhancer of angiogenesis and osteogenesis (80). MiRNA-184 targets nuclear factor I-C (NFI-C) (65) the downstream gene of RUNX2, which regulates osterix expression in osteoblast differentiation (81). MiRNA-30c targets bone gamma-carboxyglutamate protein (BGLAP) also known as osteocalcin (61), the characteristic marker of osteoblasts, and a large amount of secreted protein at the beginning of mineralization (82). Nonetheless, some miRNAs, such as miR-589-3p, -543, -2861, -185b-5p, -21 and -101 promote osteogenic differentiation (60, 64, 67, 68, 70). MiRNA-589-3p targets activating transcription factor 1 (ATF1) (68). MiRNA-543 targets the transducer of ERBB2,2 (TOB2) which suppresses osteogenic differentiation through cell cycle regulation (67). Exosome-derived miRNA-185b-5p from mechanical-strained osteocytes promote PDLSCs osteogenic differentiation by targeting phosphatase and tensin homolog (PTEN), the inhibitor of PI3k in PI3k/AKT signaling pathway (64). In addition, miR-21 and miR-101 targets periodontal ligament associated protein 1 (PLAP1), the marker which indicates an inhibition of osteogenic differentiation of periodontal ligament cells for homeostasis of periodontal tissue (60). Some miRNAs are related to PDLSCs osteogenic differentiation, but their mechanism is not yet clarified. The study by Zhou et al. demonstrated miRNA-18a and miRNA-133a level are opposite to miRNA-141 and miRNA-19a during osteogenic differentiation (83).

Collectively, there are many miRNAs involved in osteogenic differentiation. These miRNAs can be proposed in the application of tissue engineering and increase success of clinical outcomes.

Some non-coding RNAs, including circular RNAs (circRNAs) or long non-coding RNAs (lncRNAs), are known to have a collaborative role with miRNAs to regulate cell function. The following data of small non-coding RNAs emerged during the search of osteogenic differentiation of PDLSC. CircRNAs form as a miRNA and RNA binding sponge and prevent miRNA from functioning (84). lncRNAs also have a role to regulate miRNA expression by forming a sponge with transcription factors or chromatin complexes to block their interaction and functions (85). lncRNA and circRNAs were shown to be differentially expressed in osteogenic differentiation (86, 87) as they may counteract miRNAs binding on their target during cell differentiation (87). Several lncRNAs, including H19, DANCR, MALAT1, MEG3 or HOTAIR, contribute to osteogenic differentiation, or as a key regulator in various types of cells (88). Collaborative functions of these small non-coding RNAs to miRNAs function could not be neglected, but it is not in a scope of this review.

### MicroRNA of PDLSCs under inflammation conditions

Inflammation is a host reaction to trauma, infections, toxic substances, or injury aiming for tissue healing. Inflammatory responses may occur against the implanted biomaterials when regenerating the tissue as a foreign body (89). The crosstalk between immune cells such as macrophages and the stem cells can influence the regulation of bone regeneration (90). Therefore, it is important to consider the alteration of stemness and osteogenic potential of PDLSCs especially when inflamed tissue becomes a source for cell therapy. This scoping review explored how differential miRNA profile affects PDLSCs osteogenic potential under inflammation conditions. Recent studies mentioned different types of inflammation that is induced by bacterial infection such as periodontitis, mechanical force in orthodontic movement, high-glucose environment in diabetes, or smoking. In these condition, differential expression of some miRNAs (Table 2) compromises the osteogenic potential of PDLSCs (31), while some miRNAs can restore the functions (38). From these data, PDLSCs osteogenic potential appears to be altered in inflamed periodontal tissue, but the underlying molecular mechanisms remain poorly understood.

**Table 2:** MiRNAs involvement in osteogenic differentiation of PDLSC in inflammatory condition.

Inflammation	MicroRNA	Effect	Target	Reference
Periodontitis	miRNA-17	Promote	HDAC9	(91)
	miRNA-21	Promote	Spry1	(92)
	miRNA-23a	Suppress	BMPR1B	(93)
	miRNA-23b	Suppress	RUNX2	(94)
	miRNA-27a-3p	Suppress	IGF1	(95)
	miRNA-138	Suppress	Osteocalcin	(96)
	miRNA-182	Suppress	FOXO1	(97)
	miRNA-146a	Promote	NF-κB p65	(98)
	miRNA-148a	Suppress	NRP1	(99)
	miRNA-3679-3p miRNA-6747-5p	Promote	GREM-1	(100)
Force induction	miRNA-21	Promote	ACVR2B	(101)
	miRNA-195-5p	Suppress	WNT3A, FGF2, BMPR1A	(102)
Diabetes	miRNA-17	Promote	Unspecified	(103)
	miRNA-31	Suppress	Satb2	(104)
Smoking	miRNA-1305	Suppress	RUNX2	(105)

#### Periodontitis

PDLSCs collected from tissue with periodontitis may undergo osteogenic differentiation through signaling other than the canonical Wnt/β-catenin pathway. The increase of tumor necrosis factor*α* (TNF*α*) during inflammation could inhibit osteogenic potential of PDLSCs mainly through upregulating the canonical Wnt pathway (106). In TNF*α*-stimulated PDLSCs, miRNA-23b is expressed to suppress RUNX2 (94). Upon differentiation of periodontitis PDLSCs, FOXO1 gene was increased and competitively bind β-catenin to inhibit the canonical Wnt/β-catenin (107). However, miRNA-182 was overexpressed under Inflammatory conditions (97). MiRNA-182 targeted FOXO1 gene leading to decrease of osteogenic differentiation in periodontitis PDLSC (97). During infection, the bacterial lipopolysaccharide (LPS) activate host immune responses (96, 98) and NF-κB pathway to reduce the osteogenic differentiation of PDLSCs (108). In contrast, the overexpression of miRNA-146a decrease the NF-κB signaling, and rescued the osteogenic potential (74, 108). MicroRNA-146a was downregulated and IL-13 was upregulated in PDLSCs derived from periodontitis-affected teeth. Overexpression of microRNA-146a improve periodontitis by downregulating IL-13 and inhibiting the proliferation of PDLSCs derived from both periodontitis-affected teeth and healthy teeth (109). MiRNA-138 and miRNA-148a were also increased in periodontitis by LPS stimulation (96, 99), but function in contrast to miRNA-146a (98). MiRNA-138 and miRNA-148a targeted the osteocalcin promoter region and neuropilin 1 (NRP1), the member of the neuropilin family, respectively, to regulate cell proliferation, apoptosis, and differentiation (96). Therefore, expression of miRNA-138 and miRNA-148a were related to an impairment of osteogenic differentiation in PDLSCs.

In PDLSCs extracted from inflamed tissue, the miRNA involvement has been identified by comparing PDLSCs from healthy and inflamed tissue. Under inflammatory conditions, HDAC9 of the HDAC family plays a role in decreasing osteogenic differentiation capacity of PDLSCs. Consistent to HDAC9 function, PDLSCs from periodontitis tissue down regulated miRNA-17 level (91). MiRNA-17 was able to inhibit HDAC9 in inflamed PDLSCs and recover the osteogenic potential to a healthy level (91). In addition, miRNA-17 targets 3'UTR of Smad ubiquitin regulatory factor1 (Smurf1), which is another negative regulator of osteogenic differentiation. Therefore, downregulation of miRNA-17 in inflammatory microenvironments resulted in lower osteogenic differentiation by allowing the functions of HDAC9 (91) and Smurf1 (48). In addition, miRNA-27a-3p and miRNA-23a levels are upregulated in PDLSCs from inflamed tissue. MiRNA-27a-3p can target IGF1 gene which upregulates the osteogenic differentiation by activation of PI3K/Akt signaling pathway (95), while MiRNA-23a targets bone morphogenetic protein receptor 1B (BMPR1B) (93). Increase of both miRNA-27a-3p and miRNA-23a in inflamed PDLSCs resulted in impaired osteogenic activity of PDLSCs. In contrast, exogenous miR-3679-5p and miR-6747-5p rescued osteogenic ability of the PDLSCs from inflamed tissue by targeting GREM-1, the BMP signaling inhibitor, and therefore allowed the BMP-dependent osteogenic differentiation (100). In addition, miRNA-21 level is downregulated, but Spry1, the negative regulator of ERK-MAPK pathway, is up-regulated (92). As miRNA-21 directly targeted Spry1, therefore overexpression of miRNA-21 can rescue the impair osteogenic differentiation of PDLSC under TNF*α* stimulation.

#### Force-induced inflammation

PDLSCs osteogenic differentiation ability changes under force-induced inflammation during orthodontic tooth movement. The inflamed PDL tissue under compressive or tensile force demonstrates an alteration of blood flow and accumulation of biological molecules such as neurotransmitters, cytokines, and arachidonic acid metabolites to mediate inflammatory reactions (110). The tensile force stretches PDL and favors osteogenic differentiation (101, 102, 111), while the compressive force enhances bone resorption. The role of miRNAs, such as miRNA 195-5p, miRNA-21, miRNA-29, in these conditions by microarray have been reported (101, 102), and some miRNAs are related to osteogenic potential of PDLSCs. Under a cyclic application of tensile force, miRNA 195-5p, targeting Wnt3A, fibroblast growth factor2 (FGF2) and BMP receptor 1 A (BMPR1A), was downregulated in the periodontal tissue of the mouse model (111). The positive regulation of these molecules therefore promotes osteogenic differentiation by RUNX2 and OSX activation (111). Consistently, miRNA-21 was upregulated resulting in increase of osteogenic differentiation in PDLSCs along with downregulation of activin receptor IIB (ACVR2B), in the same tensile force condition. MiRNA-21 targeted ACVR2B, a transmembrane receptor kinase for activation of activin in the TGF-β pathway which has a crucial role in cell differentiation (101). MiRNA-29 is also involved in the extracellular matrix remodeling by targeting extracellular matrix gene collagen 1A, 3A1 and 5A1. At the tension side, miRNA-29 is downregulated resulting in an increase of extracellular matrix in contrast to the compression side (112).

#### Diabetes mellitus

Diabetes mellitus is a chronic inflammation disorder that affects whole body including periodontal tissue (113). The periodontium demonstrates an accumulation of the advanced glycation end products (AGEs) in extracellular matrices in diabetic patients (113). The accumulation of AGEs leads to inflammation of the periodontal tissues (114), and disturbs PDLSC differentiation (115). Several studies have demonstrated the high-glucose environment and AGEs affecting miRNA expression in the PDLSCs culture. Stimulated with exogenous AGEs *in vitro*, PDLSC differentiation was diminished along with down-regulated miRNA-17, while transfection of miRNA-17 rescued the effect (103). In addition to miRNA-17, MiRNA-31 was also increased in the periodontal tissue of the diabetic mouse model with induced periodontitis (104). The miRNA-31 was shown to target SATB2, a special AT rich sequence binding protein, mRNA which protein regulates a chromatin structure during osteoblast differentiation (116). MiRNA-31 appeared elevated in the high-glucose environment (117), while the osteogenic differentiation of PDLSCs was inhibited (104).

#### Smoking

Smoking and nicotine are also the external stimuli that can negatively affect tissue homeostasis. It was reported that smoking delayed wound healing by inhibiting stem cells (118, 119). PDLSCs cultured from smokers demonstrated a substantial reduction of cell proliferation rate, migration capabilities, and osteogenic differentiation (105). Another *in-vitro* study demonstrated treatment of 1.0 mM nicotine reduced osteogenic gene expression and mineralization of PDLSCs in culture (120), while miRNA-1305 and miRNA-18b were upregulated in contrast to downregulation of miRNA-3198 (105). As RUNX2 was targeted by miRNA-1305, the miRNA-1305 inhibitor resolved the inhibitory effect of nicotine on osteogenic differentiation in PDLSCs culture (26).

Interestingly, miRNA-17 and miRNA-21 might play a role in both physiologic and inflammatory conditions, but they act contradictory by targeting different mRNAs. As a result, the expression of miRNA-17 and miRNA-21 alternately affects different signaling pathways in physiologic or inflammatory conditions. Reduction of HDAC9 by miRNA-383-5p in physiologic (57) and miRNA-17 (91) in periodontitis PDLSCs promotes osteogenic differentiation. While reduction of RUNX2 by miRNA-218 in physiologic (66) and miRNA-23b in TNF-α stimulated PDLSCs (94) (Sun et al., 2021) and miRNA-1305 in nicotine-stimulated PDLSCs (26) suppress osteogenic differentiation. Reduction of Osteocalcin by miRNA-30c in physiologic (61) and miRNA-138 in LPS-stimulated PDLSCs (96) also suppress osteogenic differentiation.

### Current application of stem cells in orofacial bone tissue engineering

Although stem cell therapies have demonstrated regenerative potential for the treatment of periodontal defects consistently in the preclinical studies (5), the benefit of stem cells remains unclear in clinical trials (121). To summarize the potential of PDLSCs therapy, and how miRNAs can improve PDLSCs properties will be beneficial for clinical application. In some studies, stem cells from different origins have been used in combination with bone scaffold to enhance bone healing (122). But another study showed no benefits of adding stem cells compared to bone graft material alone (123). The challenge remains in maintaining desired PDLSC characteristics at the surgical sites where it could be affected by local environment and immune reaction. In a diabetic rat model, bone regeneration was affected by the ROR*α* macrophage which is compromised in hyperglycemic microenvironment (124), suggesting that the inflammatory environment at recipient sites could hamper the osteogenic differentiation of PDLSCs at the surgical site. In this case, bioactive molecules like miRNAs, may improve the results of stem cell therapy by upregulating osteogenic genes (125).

Despite the challenges, the therapeutic benefits of PDLSCs may be attributed to its exosomal secretion, particularly relating to their osteogenic differentiation, for a cell-free therapy. A recent study has shown that exosome-loaded collagen sponge enhanced periodontal regeneration in a rat model without detectable adverse effects. The other study (126) demonstrated that exosome-derived miRNA-17 from nondiabetic-conditioned bone-marrow stem cells can rescue osteogenesis and bone regeneration in rats with type 2 diabetes mellitus. Thus, exosomal content could influence their neighboring cells via a paracrine mechanism (127). The recent study identified apoptotic bodies derived from mesenchymal stem cells that are enriched with miR-223-3p (128). Targetscan and luciferase activity predicted Itgb1 as a target of miR-223-3p, which inhibited osteoclast differentiation and alveolar bone resorption. When these apoptotic bodies were engulfed by pre-osteoclasts, the exosomal miR-223-3p attenuated osteoclast differentiation and bone resorption (128). Moreover, the mir338 cluster was enriched in gingival tissues of patients with chronic periodontitis and a ligature-induced periodontitis mouse model (129). Mir338 appeared to contribute to macrophage polarization and osteoclastogenesis in periodontal tissue. Thus, an attenuation of alveolar bone loss with ligature was observed in the mir338 knockout mice. Whereas the administration of miR-338-3p antagomir prevented alveolar bone loss from periodontitis. Similar studies (130, 131) suggested that PDLSCs could release soluble signaling molecules in condition mediums that alter the immune microenvironment, such as macrophage polarization, to enhance periodontal regeneration. Collectively, these studies suggested the regulatory role of PDLSCs-derived exosomes that can be applied in a cell-free approach. However, the active molecules such as those miRNAs or exosomal contents need to be properly extracted and handled with the delivery system (125). Recently, several nano-delivery systems have been suggested for miRNA therapy (132, 133). For example, the injectable nanofibrous spongy microspheres were proposed as a drug delivery vehicle (134). The system is composed of multiple biological materials carrying bioactive molecules such as IL-2/TGF-β and miR-10a to locally recruit and stimulate regulatory T cell differentiation. This system also proposed to establish a desirable microenvironment and rescue periodontal bone loss in a mouse model.

## Conclusions

This scoping review aimed to describe the differential miRNA functions in osteogenic differentiation of PDLSCs, and analyze the potential of PDLSCs and miRNAs therapy in bone regeneration. The data suggested that functions of PDLSCs support new bone induction. Periodontal and orofacial bone regeneration increases along with osteogenic differentiation of transplanted PDLSCs. Nonetheless, harvested PDLSCs may demonstrate differential, sometimes unpredicted, characteristics therefore cell sources and conditions should be carefully considered for clinical application. Osteogenic differentiation of PDLSCs is also regulated through various pathways including canonical Wnt/β-catenin, Smad, MAPK, HDAC, and other pathways that involve miRNA functions. PDLSCs were shown be influenced by local factors, therefore its osteogenic potential has variation according to microenvironment at the recipient sites such as bacterial infection, force-induced tissue inflammation, smoking, or hyperglycemic condition. Tissue inflammation tend to hamper osteogenic potential of PDLSCs. Thus, the use of cells from hopeless teeth, or cells transplanted in inflamed condition, could be compromised in PDLSCs therapy. Nonetheless, emerging from the search is the exosomal-derived miRNAs released from PDLSCs that appeared to be key molecules of PDLSCs function during bone regeneration. In this scoping review, the regulatory functions of miRNAs as a bioactive molecule are remarkable in both *in vitro* and *in vivo* model. Most studies indicate the benefits of miRNAs, or the exosomal contents, derived from PDLSCs, but also accept the limitation of using PDLSCs in clinical application. Therefore, the data enlightens miRNAs as the alternative approach, or an adjunct to cell therapy. Further studies of the delivery systems will be required to efficiently facilitate the use of miRNAs or PDLSCs-derived exosome in clinical application.
