# Supplementary material for: MicroRNA functions in osteogenic differentiation of periodontal ligament stem cells: a scoping review
Source: Front Oral Health. 2025 Jan 31;6:1423226. doi: 10.3389/froh.2025.1423226 (PMC11825769; doi:10.3389/froh.2025.1423226)
Supplement: Supplementary file 1 [file Table1.docx]

**Supplement Table 1** **Inclusion criteria, exclusion criteria, and the search terms**

| **PICo tool** | **Inclusion criteria** | **Exclusion criteria** | **Search Terms** |
| --- | --- | --- | --- |
| **P - population** | Human PDLSCs originated from healthy or inflamed periodontal ligament | Other types of human stem cells such as bone marrow-derived stem cells etc. | **Google scholar: allintitle: osteogenic differentiation periodontal ligament cells miRNA OR microRNA OR PDLSCs OR PDLSC**  **PubMed: periodontal ligament stem cells miRNA osteogenic differentiation**  **EBSCO: Boolean/Phrase: Periodontal ligament stem cells and osteogenic differentiation** |
| **I – phenomenon of interest** | MiRNA-related osteogenic differentiation, or stemness, demonstrated in either in-vitro or in-vivo studies | Osteogenic differentiation induced by other bioactive molecules, such as BMP. |  |
| **Co - context** | -Healthy tissue  -Periodontitis or bacterial-induced inflammation  -Orthodontic-induced, smoking, or diabetic-induced inflammation | n/a |  |
